# pH-Dependent Gelation of a Stiff Anionic Polysaccharide in the Presence of Metal Ions

**DOI:** 10.3390/polym12040868

**Published:** 2020-04-10

**Authors:** Andrey V. Shibaev, Dmitry A. Muravlev, Aleksandra K. Muravleva, Vladimir V. Matveev, Anatoly E. Chalykh, Olga E. Philippova

**Affiliations:** 1 Physics Department, Lomonosov Moscow State University, 119991 Moscow, Russia; phil@polly.phys.msu.ru; 2 Department of Gas Chemistry, Gubkin Russian State University of Oil and Gas, 119991 Moscow, Russia; mit89-angel@yandex.ru (D.A.M.);; 3 Frumkin Institute of Physical Chemistry and Electrochemistry, Russian Academy of Sciences, 119071 Moscow, Russiachalykh@phyche.ac.ru (A.E.C.)

**Keywords:** polysaccharide, xanthan, cross-link, rheometry

## Abstract

Cross-linking of polysaccharides by metal ions provides polymer gels highly required by industrial applications. In this article, we study the rheological properties and microstructure of solutions of a stiff anionic polysaccharide xanthan cross-linked by chromium (III) ions, and we demonstrate that their properties are highly sensitive to the preparation pH. Stable gels are obtained in a wide range of pH from 2.4 to 7.8. The maximum elastic modulus is observed for the gels made at pH 6.3, and by freeze-fracture transmission electron microscopy it is shown that they are characterized by the most dense network structure. However, out of this pH interval, no gelation is observed. At low pH (< 2.4) it is due to high protonation of carboxylic groups of xanthan preventing their interaction with chromium ions, and to the disappearance of oligomeric ions, which are effective in cross-linking. At high pH (> 7.8) the absence of gelation is caused by the transformation of reactive chromium ions into insoluble chromium hydroxide. At the same time, for the gels initially formed at pH 6.3, subsequent change of pH to strongly acidic (1.4) or basic (8.9) medium does not affect appreciably their properties, meaning that chromium cross-links are stable once they are formed. These observations open a reliable route to produce polysaccharide gels with required mechanical properties in a wide pH range where they initially cannot be prepared. It is also shown that the increase of pH to 6.3 of the initially ungelled solution prepared at pH 1.5 results in gelation. This effect offers a facile way for delayed gelation of polysaccharides, which is especially required by oil industry.

## 1. Introduction

Hydrogels of natural polyelectrolytes draw much attention due to the combination of interesting rheological properties and commercial availability of these polymers [1,2,3,4,5]. Stiff polysaccharides are of particular interest, since they can form networks with rather high viscoelasticity at very low polymer concentrations (around 0.05–0.1%) [6]. Due to this, they are widely used as thickeners in various industrial applications, e.g., in the production of consumer goods and food industry [7,8], materials for biomedical applications [9,10] etc. Their distinct application is the preparation of fracturing fluids for oil industry [11]. Such fluids consist of proppant particles (sand or ceramics) suspended in a gel or a viscoelastic medium, and are used for enhancement of the oil recovery process [12]. Currently, polymer [13] or surfactant [14,15] viscoelastic solutions are used as the main component of the fracturing fluids. However, the properties and microstructure of stiff polysaccharide gels are much less investigated than those of flexible or semiflexible polysaccharides used in oil recovery (including guar, cellulose and their derivatives).

**Figure 1:**
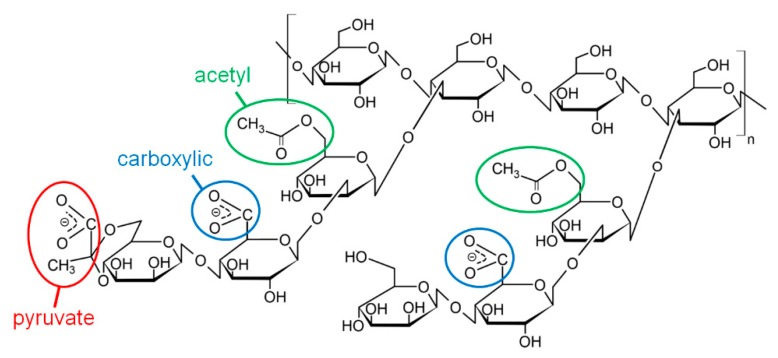
Chemical structure of xanthan (two monomer units with and without pyruvate group are shown).

One of the most widely used stiff natural polymers is xanthan, which is a bacterial polysaccharide produced by aerobic bacteria *Xanthomonas campestris*. Xanthan macromolecules (Figure 1) consist of a cellulose backbone containing β-(1-4)-D-glucose units, and trisaccharide side chains attached to every second glucose unit [8]. The side chains contain two mannose residues, one of which may contain a carboxylic group, and one glucuronic acid residue, which may contain a pyruvate group [16], making xanthan an anionic polysaccharide. Native xanthan macromolecules have a secondary structure of double helix [17]. Due to this, xanthan has one of the highest values of persistence length among natural polymers, which is ca. 120 nm [18]. This imparts very interesting features to the microstructure of xanthan gels: they have a microphase separated structure with polymer-rich areas (network skeleton) formed by side-by-side aggregation of xanthan double helices [6,19]. The parts of the skeleton inside one mesh are very rigid, but at some points they form sharp kinks due to the presence of short flexible macromolecular segments—“melted” xanthan helices, which join rigid double helix segments together. Recently, similar structure was observed for other stiff polysaccharides [20].

One of the possible ways for improvement of the mechanical properties of xanthan gels is the cross-linking of polymer chains by metal ions [21]. Xanthan macromolecules can be cross-linked by various multivalent ions (Cr^3+^, Fe^3+^, Al^3+^, Zr^4+^, etc.) [22,23], which interact with carboxylic groups of different macromolecules. The mechanical properties of the gels depend on the strength of such cross-links. For various ions, different values of the cross-linking enthalpy have been reported [22,24]. One of the particularly interesting cases is the cross-linking of polyelectrolytes by Cr^3+^ ions [21,25], since the nature of such cross-links is mainly not electrostatic interaction between the Cr^3+^ cation and –COO^−^ group, but the formation of a stronger chemical bond [26]. At the same time, it is reported in the literature that Cr^3+^-cross-linked xanthan hydrogels can restore their properties after disruption by strong mechanical shear [27], suggesting that the cross-linking process may be reversible. However, the detailed mechanism of cross-link formation, as well as the influence of different parameters (pH, presence of low molecular weight salt, which screens the electrostatic interactions, polymer concentration, etc.) on cross-linking, are not well understood. For instance, the data concerning the effect of pH on xanthan gelation are rather contradictory [22,28,29]. Most works were focused on the effect of pH on the gelation rate. For instance, in the work [28] it was shown that the initial gelation rate of xanthan/Cr^3+^ systems is inversely proportional to the H^+^ concentration. It reaches a maximum at pH ~ 6.5, but decreases at higher pH due to precipitation of chromium. However, no explanations for this behavior on the molecular level were presented. In the work [29], a particular case was studied when pH of xanthan/Cr^3+^ system was kept constant by autotitration during gelation, and it was observed that the gelation time decreased with pH increase for different chromium salts (nitrate, triacetate or basic acetate). An explanation was related to the fact that, at different pH, various chromium species are formed, which cross-link xanthan more or less efficiently. In particular, authors assumed that, in the studied pH range of 2.5–5, Cr(OH)(H_2_O)_5_^2+^ and Cr(OH)_2_(H_2_O)_4_^2+^ hydroxides are formed with increasing pH, and they react faster with polymer carboxylic groups than Cr(H_2_O)_6_^3+^ ion, which is initially formed after dissolution of Cr(III) salts in water [30]. In the work [22] it was discovered that the elastic modulus of xanthan gels reaches a maximum at a certain pH values, which are different for Fe^3+^, Al^3+^ and Cr^3+^. For Cr^3+^, the elastic modulus increased with pH and its maximum value was observed at pH 5.0. It was explained by the formation of dimeric or higher oligomeric species of ions with increasing pH, which are more effective in polymer cross-linking. However, the pH range where gels are not formed was not concerned.

Therefore, this paper is aimed at the investigation of the effect of pH on the mechanical properties and microstructure of xanthan solutions in the presence of a cross-linker—chromium (III) chloride. By combining rheological measurements with the investigations by freeze-fracture transmission electron microscopy (FF-TEM), we show that at low pH xanthan macromolecules cannot be cross-linked due to the difficulty of interaction between chromium species with –COOH groups; at higher pH cross-linking occurs due to the facilitation of the interaction of ions with charged –COO^−^ groups, resulting in the formation of highly elastic networks; and, finally, at very high pH xanthan networks are not formed due to transformation of Cr^3+^ into water insoluble Cr(OH)_3_. We discover for the first time that the change of pH of the gels after their formation to strong acidic or basic values does not lead to the disruption of the networks and degradation of the cross-links. At the same time, the change of pH to ~ 6.3 of the ungelled solutions prepared at acidic conditions results in gelation due to deprotonation of –COOH groups, which may be a basis of a new application of such fluids as water-blocking agents, which form a gel after pumping into the well upon contact with formation water.

## 2. Materials and Methods

### 2.1. Materials

Xanthan gum (Ziboxan F200) of food grade was obtained from Deosen Biochemical Ltd. (Ordos, China). Its molecular weight is equal to 1,000,000 g/mol, which was estimated by viscometric measurements in our previous work [6]. The obtained value of molecular weight corresponds to the degree of polymerization of ca. 1100. The degrees of acetyl and pyruvate substitution of xanthan (molar content per repeat unit) were equal to 0.56 and 0.41, respectively, as determined by ^1^H-NMR [6]. Chromium (III) chloride hexahydrate (Sigma Aldrich, Saint louis, MO, USA, 98%), potassium chloride (Acros, Geel, Belgium, 99.8%) and sodium azide (Sigma Aldrich, Saint louis, MO, USA, 99.5%) were used as received. In order to vary the pH of the samples, potassium hydroxide (Acros, Geel, Belgium, 98%) and hydrochloric acid as a titration standard for preparation of 1 M or 0.1 M solution (ZAO Uralchiminvest, Ufa, Russia, 99%) were used. The solutions were prepared in distilled deionized water purified by the Milli-Q system (Millipore, Burlington, MA, USA).

### 2.2. Preparation of Solutions

First, stock aqueous solutions of xanthan (0.5–2 wt %), chromium chloride (1 wt %) and potassium chloride (20 wt %) were prepared. Xanthan stock solution was made by dissolving its powder in distilled water containing NaN_3_ (final concentration in the samples: 3 mM) as a bacteriostatic agent under gentle stirring during 24 h. Stock solution of cross-linker (chromium chloride) was prepared by stirring for 5–10 min; then it was left for 2 days in order to allow the hydrolysis to proceed; the final stock solution had a violet-green color. Xanthan solutions in the absence of CrCl_3_ at different pH were prepared by mixing the appropriate quantities of xanthan stock solution and 1 M solutions of KOH or HCl, followed by magnetic stirring for 2 h. Xanthan solutions and gels in the presence of CrCl_3_ at different pH were prepared in the following way. First, xanthan stock solution was mixed with different amounts of 1 M solutions of KOH or HCl (and KCl stock solution, if necessary) under stirring for 2 h. At this stage, pH of the solutions was higher than the final pH of the samples. Then, the stock solution of CrCl_3_ was added, and the solutions were mixed by vigorous stirring for 10 min. The addition of CrCl_3_ lowered the pH to the final value. After that, the solutions were left for cross-linking to proceed, and were inspected 2 weeks after preparation.

The effect of pH on the already prepared cross-linked xanthan gels was studied by adding a small amount of acid or alkali (150 μL of 5M HCl or 4.5 μL of 5 M KOH) to 4 mL gels initially obtained at pH 6.3. Then, the gels were very gently shaken overnight in order to allow the acid or alkali to diffuse into the gel. Uniform distribution of KOH or HCl was proven by measuring pH at different points of the gel. The influence of pH on the uncross-linked xanthan solution was investigated by adding 138 μL of 5 M KOH to a 4 mL solution initially obtained at pH 1.5.

### 2.3. Rheometry

Rheological measurements were performed using the rotational rheometer Physica MCR 301 (Anton Paar, Graz, Austria). The details of the measurements are described elsewhere [31,32,33]. For the experiments with solutions, which did not show gel-like behavior (in the absence of cross-linker, or in the presence of cross-linker at low and high pH) double gap coaxial cylinders with 19.14 mm average radius and 55 mm height were used. For the experiments with the gels, the samples with 25 mm diameter and 6–8 mm height were synthesized. Plate-plate geometry of the measurement cell with rough surface was used to eliminate slipping of the sample. The diameter of the upper moving plate was 25 mm. Temperature was controlled by Peltier elements and set at 20.00 ± 0.05 °C. A specially constructed vapor lock was used to prevent solvent evaporation from the sample. From oscillatory shear measurements, frequency dependences of the storage (G’) and loss (G”) moduli were determined in the angular frequency (ω) range of 0.01–50 s^−1^. All measurements were made in the linear viscoelastic regime, where the amplitude of deformation was in the range 1–5% and storage and loss moduli were independent of deformation.

### 2.4. Freeze-Fracture Transmission Electron Microscopy (FF-TEM)

FF-TEM experiments were conducted on EM-301 microscope (Philips, Eindhoven, The Netherlands). The samples for these studies were prepared as follows. The copper cell was cooled down to the temperature of liquid nitrogen. Then a small volume of solution (~ 0.1 cm^3^) was put into the copper cell and cooled down by liquid nitrogen at a very fast rate (500 K/s), which allowed it to obtain vitrified amorphous water and avoid its crystallization, preserving the native structure of the solution. After that, the samples were put under vacuum (10^−5^ torr) at continuous cooling with liquid nitrogen, and a fracture was made. The fractured surfaces were etched for 10–20 min at 10^−5^ torr at the temperature of liquid nitrogen and then replicated by spraying platinum and carbon. The cell containing this replica was unfrozen; the replica was put onto an electron microscopy copper grid, washed several times, dried, and examined with a microscope.

In order to construct the histograms of the object sizes and mesh sizes of the network at the FF-TEM images, they were processed by the software ImageJ [34]. From FF-TEM images of the networks the mesh size was estimated as the cube root of the sum of three values of the diameter of each mesh measured at different directions.

## 3. Results and Discussion

In this article, the effect of pH on the gelation of an anionic polysaccharide xanthan in the presence of trivalent metal ions (Cr^3+^) was studied. The concentration of xanthan was 0.1 or 1 wt % (10^−3^ or 10^−2^ monomol/l). The concentration of 0.1 wt % corresponds to the semidilute unentangled regime, while 1 wt % corresponds to the semidilute entangled regime [6]. In both these regimes, xanthan gels can be obtained by cross-linking the macromolecules by chromium ions [6]. Cross-linking proceeds by the interaction of multivalent cations with carboxylic groups of different macromolecules. Xanthan contains carboxylic groups at two different positions: 1) attached to the second mannose unit of the side chain and 2) as a part of the pyruvate group of the glucuronic acid unit of the side chain (Figure 1). It was shown [35] that the pyruvate groups are preferentially involved in the interactions with cations, since they are located at the end of the side chains, and steric hindrances do not prevent their close contact with neighboring macromolecules; however, some carboxylic groups of the mannose units may also be involved in cross-linking. In this work, the concentration of cross-linker CrCl_3_ was chosen so that the molar ratio [CrCl_3_]/[xanthan monomer units] was equal to 1.

### 3.1. Effect of Preparation pH on the Mechanical Properties

**Figure 2:**
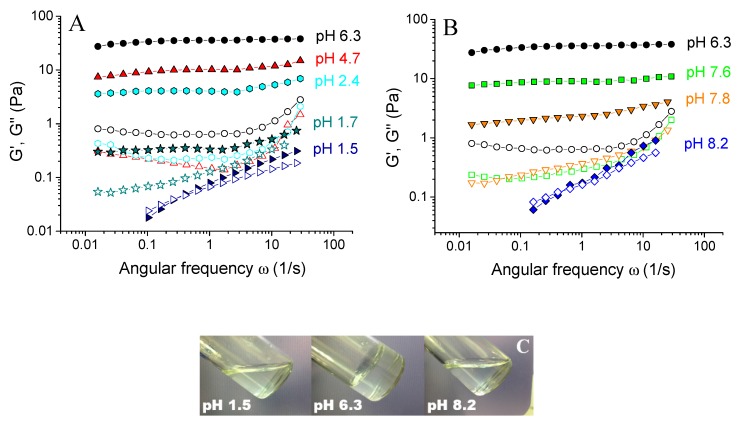
Frequency dependences of storage Gʹ (filled symbols) and loss G” (open symbols) moduli for 0.1 wt % xanthan aqueous solutions in the presence of 0.017 wt % CrCl_3_ (molar ratio [CrCl_3_]/[xanthan monomer units] = 1) prepared at different pH: 1.5 (tilted triangles), 1.7 (stars), 2.4 (diamonds), 4.7 (triangles), 6.3 (circles), 7.6 (squares), 7.8 (reverse triangles) and 8.2 (diamonds), at 20 °C (**A** and **B**). Photographs of 0.1 wt % xanthan aqueous solutions in the presence of 0.017 wt % CrCl_3_ prepared at different pH (**C**).

First, the effect of preparation pH on the rheological properties of xanthan/CrCl_3_ aqueous solutions was studied. It is seen from Figure 2 that they show gel-like behavior in a rather wide range of pH from 1.7 to 7.8: the storage modulus G’ was higher than the loss modulus G’’ in the whole range of frequencies studied, and G’ was nearly independent of frequency and shows a rubbery plateau. This was evidence for the formation of cross-linked xanthan network in the whole volume of the sample. However, at very low (1.5) or at very high pH (8.2) the samples lose elastic properties, which was evidenced by the disappearance of the plateau at G’(ω) dependence, and by the presence of cross-over point of G’(ω) and G’’(ω). Such a change in the rheological behavior manifested in the visual appearance of the samples, which behaved like gels and did not flow at intermediate pH, but are liquid-like at low or high pH (Figure 2C).

**Figure 3:**
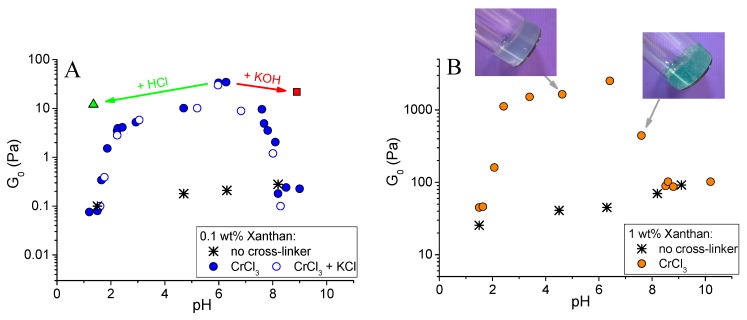
Dependence of the plateau storage modulus G_0_ on pH for 0.1 wt % (**A**) and 1 wt % (**B**) xanthan aqueous solutions in the absence of cross-linker (asterisks), in the presence of CrCl_3_ at molar ratio [CrCl_3_]/[xanthan monomer units] = 1 (filled circles), and in the presence of the same CrCl_3_ concentration and 4.75 wt % KCl (open circles). A triangle in Figure 3B corresponds to the gel obtained by the addition of 150 μL of 5 M HCl to the 4 mL gel initially prepared at pH 6.3; and a square corresponds to the gel obtained by the addition of 4.5 μL of 5 M KOH to the 4 mL gel initially prepared at pH 6.3. The values of G_0_ for liquid samples, which do not have a plateau at the dependence G’(ω), were taken at ω = 1 s^−1^.

**Figure 4:**
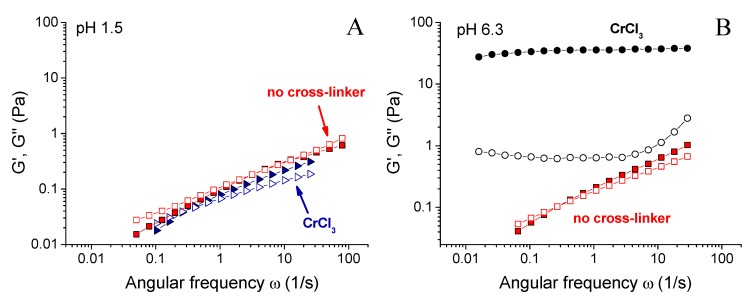

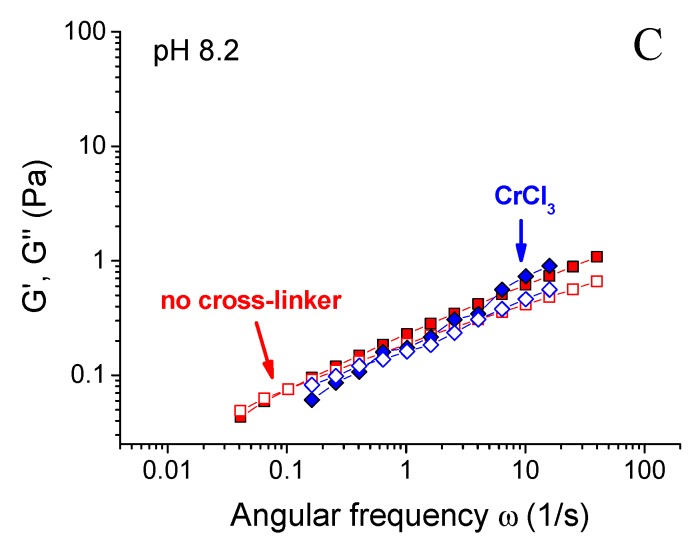
Frequency dependences of storage Gʹ (filled symbols) and loss G” (open symbols) moduli for 0.1 wt % xanthan aqueous solutions in the absence of the cross-linker (squares) and in the presence of 0.017 wt % CrCl_3_ corresponding to the molar ratio [CrCl_3_]/[xanthan monomer units] = 1 (other symbols) prepared at pH 1.5 (**A**), 6.3 (**B**) and 8.2 (**C**) at 20 °C.

Figure 3 shows the dependence of the plateau storage modulus G_0_ of the gels on pH. It can be seen that the largest value of G_0_ was observed at pH 6.3. At this pH, G_0_ for the gel with CrCl_3_ was 2 orders of magnitude higher than for the same system in the absence of the cross-linker. Figure 4B compares the rheological properties of the cross-linked and uncross-linked systems at pH 6.3, and shows that the later one behaved as a viscous liquid. The addition of CrCl_3_ to a semidilute xanthan solution results in the formation of a gel with a cross-linked network.

When pH was decreased below 6.3, the drop of the storage modulus was seen. First, only a slight 10-fold decrease of the storage modulus of the gels was observed (Figure 3A), but it stayed much higher than for the solutions in the absence of CrCl_3_. It means that the cross-linked xanthan network persisted in this range. Below a critical pH value of ~ 2.4, a drastic drop of G_0_ occurred, accompanied by a transition from a gel into a liquid (Figure 2). At pH 1.5, the rheological properties of the solutions with and without added CrCl_3_ were very close (Figure 4A), meaning that below pH 2.4 a cross-linked xanthan network was gradually disrupted, and at pH 1.5 xanthan macromolecules were no longer cross-linked.

When pH was increased above 6.3, there was a steep decrease of G_0_ by more than 2 orders of magnitude (Figure 3), also resulting in the disruption of the network and transformation of a gel into a liquid. At pH 8.2, the dependences G’(ω) and G’’(ω) for cross-linked and uncross-linked systems almost coincide (Figure 4C), meaning that cross-linking was no longer effective at these pH.

Thus, there are two critical pH values (2.4 and 6.3) corresponding to the changes in the mechanical behavior of the gels. They are related to the structure of chromium species formed in water and to the nature of their interaction with carboxylic groups. In aqueous solutions, Cr^3+^ ions coordinate six water molecules and exist in the state Cr(H_2_O)_6_^3+^, which is the predominant species at low pH < 2 [36]. At higher pH, their hydrolysis proceeds, leading to the formation of hydroxides according to the reaction [26]:

Cr(H_2_O)_6_^3+^ → Cr(OH)_n_ (H_2_O)_6−n_^(3−n)+^ + nH^+^

The fraction of each hydroxide depends on pH. Similar or different ion species formed at a certain pH can complex with each other and form oligomers, and their fractions also change with pH [36].

Cross-linking of carboxylic groups by different chromium species proceeds via a ligand-exchange reaction—coordinated water molecules are replaced by –COO^−^ groups [37]. It results in the fact that the cross-links have a mostly coordination covalent nature [38], and once they are formed, they are stable and do not continuously break and recombine, which is confirmed by high values of their activation energy reported in the literature −42 kJ/mol [22], and is consistent with the fact that the dependences G’(ω) and G’’(ω) do not intercept (Figure 2A), meaning the absence of relaxation associated with the cross-links dynamics.

Let us now consider the lower critical pH value of 2.4, which may be attributed to two factors. The first reason is that chromium monomeric species are not effective in cross-linking of carboxylic groups [22], which may be related to their small size, resulting in inability to coordinate two carboxylic groups [26]. On the contrary, olates (e.g., dimeric, trimeric and tetrameric oligomers) can cross-link xanthan [26]. When pH is below ca. 2, only monomeric Cr(H_2_O)_6_^3+^ ions exist in the solution. Above pH 2, dimeric (Cr_2_(OH)_2_^4+^) and then trimeric (Cr_3_(OH)_4_^5+^) species are formed, and cross-linking may occur. The second reason is that, at these pH values, the transformation of protonated –COOH units to deprotonated –COO^−^ form occurs [39]. Indeed, pK_a_ of xanthan was found to be around 2.6 [40]. At lower pH, more carboxylic groups of xanthan progressively become protonated, which impedes their interaction with chromium ions, since they cannot displace protons of –COOH groups. An example of acrylamide-acrylic acid copolymer was treated in the literature [41], which has a higher pK_a_ of ca. 4.5 [42]. The authors noticed that at pH 3, when both monomeric and oligomeric chromium species are present, the amount of ionized carboxylic groups is too low, so that gelation cannot occur. Consequently, at pH higher than 2.4 xanthan macromolecules are cross-linked because of two factors: 1) deprotonation of carboxylic groups, which facilitates their interaction with cross-linkers, and 2) appearance of olates, which are effective cross-linkers.

Therefore, the amount of cross-links depends both on the concentration of –COO^−^ units, as well as the concentration of chromium olates. This explains the drastic drop of the mechanical properties of the gels upon the decrease of pH below the value of 2.4. Note that at pH 1.7 the system is still gel-like (Figure 2A), but this gel is much weaker than at pH 2.4. At pH 1.5 most carboxylic groups become uncharged, and no olates are present, so that the sample behaves as a solution without the cross-linker (Figure 4A).

Let us now consider the higher critical pH value of 6.3. At pH higher than ca. 6 chromium ions transform into water insoluble hydroxide Cr(OH)_3_ [43], chromium ions do not exist in the solution, and xanthan macromolecules cannot be cross-linked into a network. It results in the decrease of mechanical properties of the samples down to the values observed for uncross-linked xanthan (Figure 3 and Figure 4C).

The effect of pH on the properties of xanthan gels was similar in semidilute unentangled regime (at 0.1 wt % xanthan, Figure 3A) and in semidilute entangled regime (at 1 wt % xanthan, Figure 3B): the critical pH values (2.4 and 6.3) were equal for both xanthan concentrations. Though 1 wt % xanthan solutions behave as highly viscoelastic solutions even in the absence of the cross-linker [44], their elasticity is greatly enhanced by the addition of Cr^3+^ cross-linker in the pH range 2.4–6.3. The elastic modulus at pH 6.3 exceeds 2.5 kPa. For 1 wt % xanthan, the formation of insoluble chromium (III) hydroxide at high pH is confirmed by the visual appearance of the gels: they are bluish at pH < 6 due to the presence of unreacted excess Cr^3+^ ions, but become green at pH > 6 due to the presence of Cr(OH)_3_.

pH-dependent cross-linking of xanthan is not affected by the addition of low molecular weight salt. It is seen from Figure 3A that the shape of the curve and the values of the plateau storage modulus of the samples in the absence of salt and in the presence of 4.75 wt % KCl coincided in the whole pH range. It means that the binding of chromium ions to the carboxylic groups did not change when electrostatic interactions were screened, and indicates that the nature of interaction between the ions and –COO^−^ groups was not electrostatic.

Therefore, in a rather wide pH range (2.4 – 6.3) xanthan forms gels with high elasticity due to cross-linking of the –COO^−^ groups by chromium ions. A dramatic change of mechanical properties of the gels outside this pH range was observed, which was due to two effects. First, at low pH (< 2.4), carboxylic groups of xanthan became protonated, and at the same time olate chromium species (which effectively cross-link xanthan) disappeared. Second, at high pH (> 6.3) chromium ions were transformed into Cr(OH)_3_, which did not contribute to the cross-linking. Both these effects induced a strong decrease of the mechanical properties and transformation of gels into liquids.

### 3.2. Effect of Preparation pH on Microstructure

**Figure 5:**
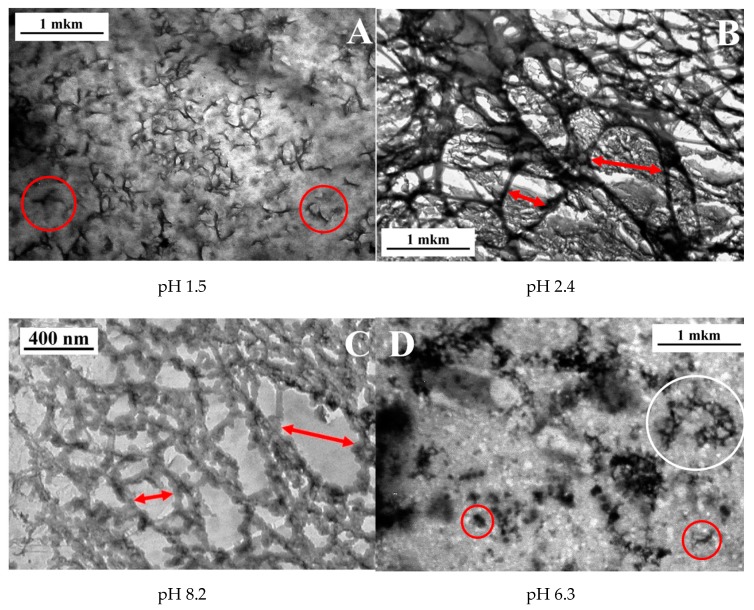
FF-TEM micrographs for 0.1 wt % xanthan aqueous solutions in the presence of 0.017 wt % CrCl_3_ (molar ratio [CrCl_3_]/[xanthan monomer units] = 1) prepared at different pH: 1.5 (**A**), 2.4 (**B**), 6.3 (**C**) and 8.2 (**D**) at 20 °C.

**Figure 6:**
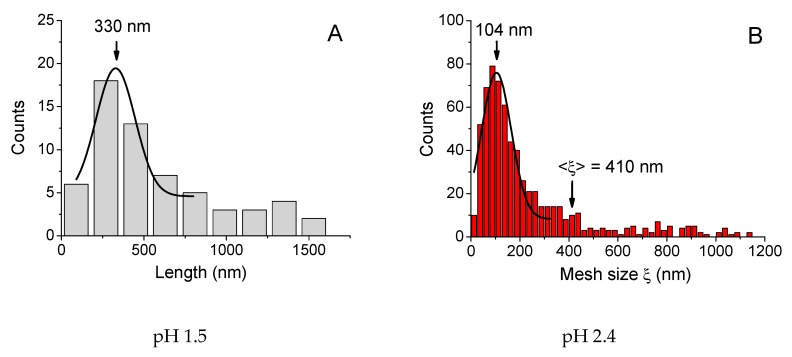

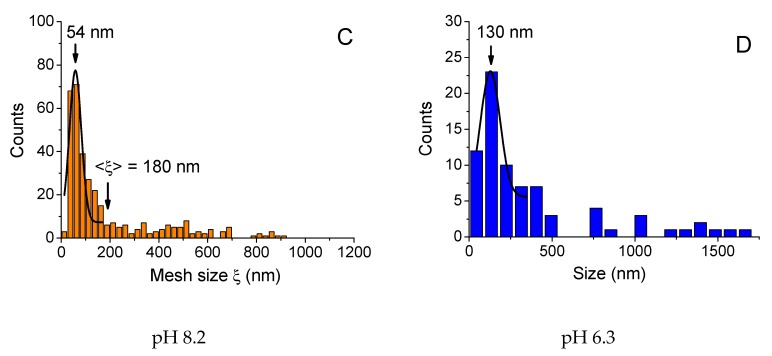
Distributions of sizes of objects (**A**, **D**) and mesh sizes of the network (**B**, **C**) obtained from FF-TEM micrographs for 0.1 wt % xanthan aqueous solutions in the presence of 0.017 wt % CrCl_3_ (molar ratio [CrCl_3_]/[xanthan monomer units] = 1) prepared at different pH: 1.5 (**A**), 2.4 (**B**), 6.3 (**C**) and 8.2 (**D**) at 20 °C. 3.3. Change of pH after Preparation

In order to get deeper insight into the reasons for the change of mechanical properties of xanthan/CrCl_3_ solutions at different pH, the rheological data were correlated with the microstructural investigations by freeze-fracture transmission electron microscopy (FF-TEM). The corresponding micrographs of the solutions are presented in Figure 5. At low pH 1.5, corresponding to the low elasticity and liquid-like behavior of the samples, some individual or partially connected elongated objects are seen in Figure 5A (marked by red circles). The mean length of individual objects was equal to 330 nm (Figure 6A), but some much larger objects (up to ca. 1–2 μm) were also present. One can suggest that all these objects correspond to single or partially overlapping xanthan macromolecules. Indeed, in the double-helix state, they have a mean contour length of several hundreds of nanometers and a persistence length of 120 nm [18], forming rigid “rod-like” objects. At pH 1.5 cross-linking of xanthan by chromium does not occur due to the fact that carboxylic groups are protonated, and, therefore, the microstructure of the sample is similar to that of xanthan solution in the absence of CrCl_3_. Indeed, no well-developed network was seen at the micrograph, which was in accordance with the fact that at 0.1 wt % the solution is in a semidilute unentangled regime.

At pH 2.4, which corresponds to the onset of high elasticity of xanthan/CrCl_3_ gels (Figure 3A), a well-developed network was seen (Figure 5B). The network had a microphase separated structure with a backbone formed by aggregated xanthan macromolecules, cross-linked together by chromium ions [6], and coexisting very large and small meshes. Histogram of mesh sizes (Figure 6B) shows that the size of larger meshes was on the order of hundreds of nanometers, while the average size of smaller meshes was 104 nm. At pH 6.3, corresponding to the maximum elasticity of the gels, a similar network structure was seen (Figure 5C). In accordance with the increase of the elastic modulus, the network became denser: the mean mesh size (calculated over all meshes) was reduced from 410 (pH 2.4) to 180 nm (pH 6.3), and the mesh size corresponding to the maximum of distribution (which represents the mean size of small meshes) decreased from 104 to 54 nm (Figure 6C).

In order to connect microstructural and mechanical properties, the mesh size ξ was estimated from the rheological data by using the theory of MacKintosh et al. [45] for entangled solutions of semiflexible polymers with the following expression for the storage modulus G_0_:

(1) $$G_{0}\approx \frac{k_{B}{T\;l}_{p}^{7/5}}{\xi^{22/5}}$$

where k_B_ is the Boltzmann constant, T the temperature and l_p_ the persistence length. The results given in Table 1 show that the mesh size calculated from G_0_ perfectly coincides with the mean size of the small meshes, which, in accordance with our previous results [6], shows that the smaller meshes contribute mostly to the elastic response and the storage modulus of the network.

Finally, at pH 8.2, no network throughout the whole volume is seen (Figure 5D), which is in accordance with the low elasticity and liquid-like behavior of the samples due to the transformation of Cr^3+^ into Cr(OH)_3_. Instead, some individual “globular” objects (marked by red circles), and network “pieces” or microgels (white circle) were present. The globular objects were presumably xanthan macromolecules with disrupted double helical structure. It was shown that for xanthan in the absence of the cross-linker alkaline medium induces conformation transition from helix to coil [46]. The mean size of the globular objects (130 nm, Figure 6D) was smaller than that for xanthan rods (330 nm, Figure 6A), which might be due to intramolecular cross-linking of xanthan coils by chromium ions. The microgels may consist of several xanthan molecules cross-linked together by Cr^3+^. The appearance of such objects may be explained by the method of preparation of the samples at high pH, which includes mixing of xanthan stock solution at high pH and CrCl_3_ stock solution at low pH. Just after mixing, in some local parts pH may be low enough (pH of the CrCl_3_ stock solution was ca. 2.5) and Cr^3+^ concentration high enough, resulting in vigorous cross-linking of macromolecules inside these areas. However, such areas were quickly separated by the parts where pH was high and Cr^3+^ readily transformed into Cr(OH)_3_. Therefore, cross-linking did not occur in the whole volume of the sample, but some microstructures cross-linked at the initial stage of mixing, did not completely break up and persisted in the solution.

Therefore, upon the increase of pH, the following sequence of structures was observed: individual uncross-linked macromolecules in a semidilute regime (pH 1.5); cross-linked 3D-network (pH 2.4 – 6.3); individual macromolecules or microgels cross-linked by chromium ions (pH 8.2).

**Figure 7:**
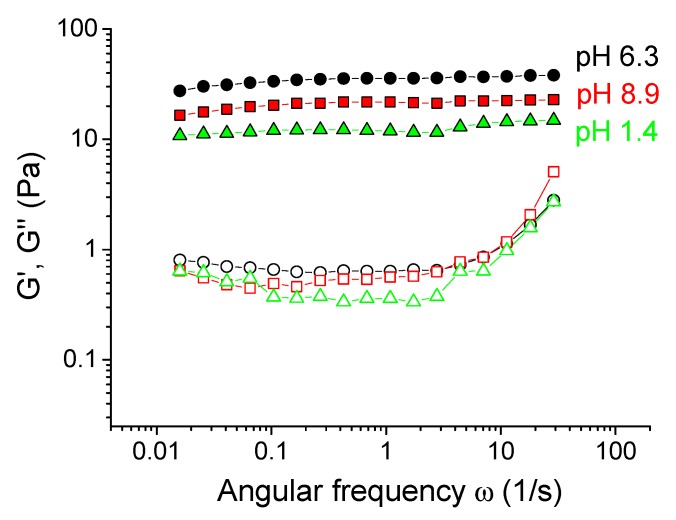
Frequency dependences of storage Gʹ (filled symbols) and loss G” (open symbols) moduli for 0.1 wt % xanthan aqueous solutions in the presence of 0.017 wt % CrCl_3_ corresponding to the molar ratio [CrCl_3_]/[xanthan monomer units] = 1: prepared at pH 6.3 (circles); obtained by the addition of 150 μL of 5 M HCl to the 4 mL gel initially prepared at pH 6.3 (triangles); obtained by the addition of 4.5 μL of 5 M KOH to the 4 mL gel initially prepared at pH 6.3 (squares).

In order to study the reversibility of xanthan cross-linking by chromium at different pH, the gels initially prepared and cross-linked at pH 6.3 (corresponding to the maximum elasticity) were taken, and their pH was changed by the addition of a small amount of acid (5 M HCl) or base (5 M KOH). It did not significantly alter the polymer concentration in the gels, but changed their pH to strongly acidic (1.4) or basic (8.9; the green and red arrows in Figure 3A represent these two routes). If the samples were initially prepared at such low or high pH values, they did not cross-link and behave as viscous liquids. However, the samples with pH “post-changed” from 6.3 remained gel-like. A weak syneresis was observed for a gel with final pH 1.4, which is explained by the fact that carboxylic groups not involved in the interaction with chromium ions become uncharged, thus reducing the counter ions pressure and the swelling degree of the gel. The mechanical properties of the gels “post-prepared” at pH 1.4 and 8.9 only slightly differed from the initial gel (Figure 7). An elastic plateau remained at the G’(ω) curve, showing that the network was not disrupted. It means that if the cross-links have been formed, they do not break upon further change of pH, and it confirms the chemical nature of the cross-links, which are coordination covalent bonds instead of ionic ones. This result opens a versatile route for preparing ion-cross-linked xanthan gels with controlled mechanical properties in a wide pH range, where they initially cannot be prepared.

**Figure 8:**
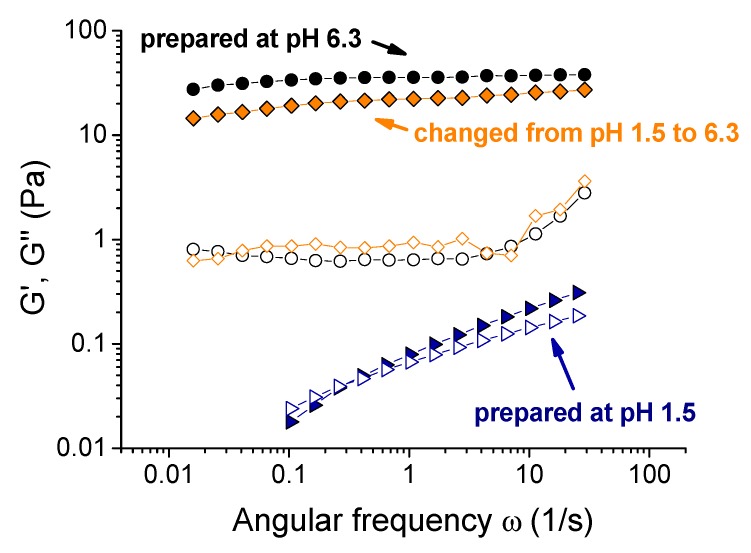
Frequency dependences of storage Gʹ (filled symbols) and loss G” (open symbols) moduli for 0.1 wt % xanthan aqueous solutions in the presence of 0.017 wt % CrCl_3_ corresponding to the molar ratio [CrCl_3_]/[xanthan monomer units] = 1: prepared at pH 1.5 (triangles); prepared at pH 6.3 (circles); obtained by the addition of 138 μL of 5 M KOH to the 4 mL solution initially prepared at pH 1.5 (diamonds).

The solution initially prepared at pH 1.5 (corresponding to the uncross-linked viscous liquid) was taken, and its pH was increased to 6.3 by a small amount of KOH. It induced a transformation from a liquid into a gel, accompanied by a drastic increase of the storage modulus (Figure 8). The rheological properties of the as-formed gel were very close to the corresponding properties of the gel initially prepared at pH 6.3. It was consistent with the fact that the gelation at pH > 2.4 was due to the formation of olate chromium species and to the deprotonation of xanthan carboxylic groups, which facilitated their interaction with chromium ions. Indeed, when the solution was initially prepared at pH 1.5, the cross-linkers were present in the fluid, but they could not act due to the difficulty for monomeric chromium to interact with carboxyl groups and to replace hydrogen. If pH was raised, −COOH groups became deprotonated, and were easily cross-linked by chromium olates formed simultaneously.

This effect is very promising for the development of polysaccharide/metal ion systems capable of “delayed” gelation. Currently, cross-linked polysaccharide gels are mostly used in the oil industry for fracturing technology [47]; however, the findings of this paper offered a possibility for a new application of such fluids as selective water-blocking agents. Indeed, a rather concentrated (for instance, 1 wt %) xanthan/cross-linker solution prepared at a pH below 1.7–1.8 did not form a gel, flowed and had limited viscoelastic properties, so that it could be pumped into the well without sufficient energy losses. When the fluid contacted the formation water, its pH increased upon dilution, and the gel was formed (for example, 1 wt % xanthan solution may be diluted 10 times, which is sufficient for the rise of pH above the gelation threshold of ~ 2.4, and xanthan concentration becomes 0.1 wt %, which is above C*, and at which xanthan forms a gel). The gel “stopper” blocked the water inflow into the well. At the same time, when the fluid contacted oil, it did not form a gel, since they are immiscible, and did not block its inflow. Therefore, such a fluid may selectively block water during oil recovery operations, which limits the amount of water extracted together with oil and reduces the necessity for further high-cost oil/water separation. Up to now, some selective water-blocking fluids based on rather complex and expensive systems (associative polymers in the presence of a gelation inhibitor [48] or magnetic polymers beads [49]) have been developed; however, to the best of our knowledge, there are no examples of such fluids based on much less expensive ion cross-linked polysaccharides. The rheological properties elaborated in this paper are of particular interest for development of such new water-blocking agents.

## 4. Conclusions

In this paper, we examined the influence of pH on the rheological behavior and microstructure of xanthan/CrCl_3_ system. We demonstrated that the rheological properties depended essentially on pH, at which the system was prepared. When the pH at preparation ranged from 2.4 to 7.8, a gel was obtained. Its storage modulus was independent of the frequency of applied stress, when the latter was varied from 0.01 to 40 s^−1^. Such behavior was attributed to the cross-linking of xanthan macromolecules by chromium ions interacting with carboxyl groups of the polymer.

However, at low pH (1.5) and high pH (8.2) the addition of CrCl_3_ did not affect the rheological properties indicating that chromium could not cross-link the macromolecules at these conditions. This behavior at low pH was explained by the absence of oligomeric chromium species, as well as by the considerable protonation of carboxylic groups, which prevented their reaction with CrCl_3_. By contrast, no gelation at high pH was due to the transformation of reactive chromium ions into insoluble form Cr(OH)_3_, which did not induce cross-linking. FF-TEM data show that in the last case some microgels were formed, whereas at pH 2.4–7.8 one could observe a well-developed network occupying the all area of observation.

Finally, when the gel was initially prepared at pH 6.3 and then the pH was decreased up to 1.5, the polymer network was not destroyed indicating that once chromium cross-links were formed, they were stable at so low a pH. On the contrary, when the solution was prepared at 1.5, and then the pH was changed to 6.3, gelation occurred. This effect offered a possibility of a new application of ion-cross-linked polysaccharide fluids as very simple water blocking agents in oil recovery.

## Figures and Tables

**Table 1:** Structural parameters obtained from FF-TEM micrographs and rheological measurements for 0.1 wt % xanthan aqueous solutions in the presence of CrCl_3_ (molar ratio [CrCl_3_]/[xanthan monomer units] = 1) prepared at different pH.

	FF-TEM				Rheometry
pH	Structure	Size of Objects (nm)	Mean Mesh Size ξ (nm)	Mesh Size at the Distribution Maximum ξ_max_ (nm)	Mesh Size ξ (nm)
1.5	Individual macromolecules or aggregates	330	-	-	-
2.4	Network	-	410	104	108
6.3	Network	-	180	54	66
8.2	Individual macromolecules or aggregates	130	-	-	-
